# Modulation of Endolysin LysECD7 Bactericidal Activity by Different Peptide Tag Fusion

**DOI:** 10.3390/biom10030440

**Published:** 2020-03-12

**Authors:** Nataliia P. Antonova, Daria V. Vasina, Evgeny O. Rubalsky, Mikhail V. Fursov, Alina S. Savinova, Igor V. Grigoriev, Evgeny V. Usachev, Natalia V. Shevlyagina, Vladimir G. Zhukhovitsky, Vadim U. Balabanyan, Vasiliy D. Potapov, Andrey V. Aleshkin, Valentine V. Makarov, Sergey M. Yudin, Alexander L. Gintsburg, Artem P. Tkachuk, Vladimir A. Gushchin

**Affiliations:** 1 N.F. Gamaleya National Research Centre for Epidemiology and Microbiology, Ministry of Health of the Russian Federation, 123098 Moscow, Russia; northernnatalia@gmail.com (N.P.A.); d.v.vasina@gmail.com (D.V.V.); alinabird@gmail.com (A.S.S.); iggrigoriev.ltb@gmail.com (I.V.G.); evgenyvusachev@gmail.com (E.V.U.); nataly-123@list.ru (N.V.S.); zhukhovitsky@rambler.ru (V.G.Z.); gintsburg@gamaleya.org (A.L.G.); artem.p.tkachuk@gmail.com (A.P.T.); 2 Lomonosov Moscow State University, 119991 Moscow, Russia; bal.pharm@mail.ru; 3 A.N. Bach Institute of Biochemistry, Research Center of Biotechnology of the Russian Academy of Sciences. 33, bld. 2 Leninsky Ave., 119071 Moscow, Russia; 4 Gabrichevsky Moscow Research Institute of Epidemiology and Microbiology, 125212 Moscow, Russian; e.o.rubalsky@gmail.com (E.O.R.); andreialeshkin@googlemail.com (A.V.A.); 5 Department of Cardiothoracic, Transplantation and Vascular Surgery, Hannover Medical School, 30625 Hannover, Germany; 6 Lower Saxony Centre for Biomedical Engineering, Implant Research and Development, 30625 Hannover, Germany; 7 State Research Center for Applied Microbiology and Biotechnology, 142279 Obolensk, Moscow Region, Russia; mikhail.fursov88@gmail.com (M.V.F.); potapovvd@mail.ru (V.D.P.); 8 Infectiology Department, I. M. Sechenov First Moscow State Medical University, 119146 Moscow, Russia; 9 Center for Strategic Planning of the Ministry of Health of the Russian Federation, 119435 Moscow, Russia; makarovvalentine@gmail.com (V.V.M.); info@cspmz.ru (S.M.Y.)

**Keywords:** endolysin, peptide tags, bactericidal activity, enzyme properties, ESKAPE pathogens

## Abstract

The use of recombinant endolysins is a promising approach for antimicrobial therapy capable of counteracting the spread of antibiotic-resistant strains. To obtain the necessary biotechnological product, diverse peptide tags are often fused to the endolysin sequence to simplify enzyme purification, improve its ability to permeabilize the bacterial outer membrane, etc. We compared the effects of two different types of protein modifications on endolysin LysECD7 bactericidal activity in vitro and demonstrated that it is significantly modulated by specific permeabilizing antimicrobial peptides, as well as by widely used histidine tags. Thus, the tags selected for the study of endolysins and during the development of biotechnological preparations should be used with the appropriate precautions to minimize false conclusions about endolysin properties. Further, modifications of LysECD7 allowed us to obtain a lytic enzyme that was largely devoid of the disadvantages of the native protein and was active over the spectra of conditions, with high in vitro bactericidal activity not only against Gram-negative, but also against Gram-positive, bacteria. This opens up the possibility of developing effective antimicrobials based on N-terminus sheep myeloid peptide of 29 amino acids (SMAP)-modified LysECD7 that can be highly active not only during topical treatment but also for systemic applications in the bloodstream and tissues.

## 1. Introduction

The spread of bacterial resistance to antibiotics has prompted the search for alternative methods to control bacterial infections. Of greatest concern to the World Health Organization (WHO) are Gram-negative pathogens that can both quickly accumulate resistance genes and cause dangerous nosocomial infections. Bacteriophage endolysins could represent a viable countermeasure [1,2]. Endolysins comprise a family of lytic enzymes that can hydrolyze the peptidoglycan of bacterial cell walls, which leads to the loss of cell wall strength and cells’ subsequent lysis. Normally, endolysins act from within the bacterial host at the end of the bacteriophage’s replicative cycle, allowing the release of viral offspring. This family of enzymes is widespread among viruses and can be found both in Gram-positive as well as in Gram-negative bacteria infecting phages. However, there are structural and functional differences between endolysins targeting Gram-positive and Gram-negative hosts [3,4]. Several “modes” of action are characteristic for endolysins, including the ability to hydrolyze either or both glycosidic and amide (including peptide) bonds [3,5].

Among the most significant advantages of using endolysins as antimicrobials is their ability to quickly and effectively lyse bacteria (even antibiotic-resistant strains and bacterial biofilms) without the development of resistance [3,6]. Thus, several endolysin molecules are currently under development [4], suggesting promising results for clinical applications. 

However, the activity of the obtained enzymes substantially depends on the method of biotechnological production. In-frame histidine tagging of proteins is a well-known and widely used approach for recombinant protein purification using metal-chelate affinity chromatography (IMAC) [7,8]. For this purpose, 5–15 histidine residues are usually added to the N- or C-terminal end of the target molecule. IMAC is very simple and compatible with purification in both native and denaturing conditions [9]. It is known that almost all protein molecules in the research phase are first expressed with a histidine tag [10]. However, there is evidence that even relatively small histidine tags, in some cases, can decrease [11], increase [12], or modify the functional activity of target molecules after fusion [10,13]. Nevertheless, researchers rarely try several tag modifications when purifying the target protein and look for alternative purification methods only if no (or few) biological effects are observed [14,15]. Most of the recombinant endolysins under investigation were purified and tested for antibacterial activity with fused histidine tags [2,6,16,17,18,19,20,21,22,23]. However, how the activity of these endolysins is modified by polyhistidine tag fusion has not been specifically studied.

Another widespread modification is endolysin fusion with antimicrobial peptides [24,25], which allows one to avoid the addition of permeabilizers like EDTA (ethylenediaminetetraacetic acid) or organic acids for the effective lysis of Gram-negative bacteria. Previously, the modification of endolysins that kill Gram-negative bacteria has been based on a fusion of a selected endolysin, and a specific outer membrane permeabilizing peptide was suggested [6]. In this case, so-called Artilysin^®^s are obtained. These peptides were designed to interfere with membrane stability because of the amino acid constitution comprising cationic and hydrophobic amino acids, giving them polycationic or amphipathic properties [2]. The best described Artilysin^®^ is Art-175, which consists of SMAP-29 (N-terminus sheep myeloid peptide of 29 amino acids) [26], Gram-negative specific endolysin KZ144 [19], and 6-His at the C-end of the molecule. It has been demonstrated that SMAP-29 fusion can significantly improve the bactericidal activity of endolysin and make it especially efficient against Gram-negative bacteria, including multidrug-resistant strains [6]. The structure and function of SMAP-29 has been extensively studied [27]. It has a minimum inhibitory concentration (MIC) at 0.3–5.2 µM for Gram-positive and 0.1–9.8 µM for Gram-negative bacteria [27,28]. However, MICs greater than 30 µM were also reported. Reasonable modifications of SMAP-29 resulted in the production of a peptide with improved antimicrobial activity and without hemolytic activity, in general for modified peptides MIC values also increased [29]. Previously, we obtained a fusion of endolysin L-KPP10 with a shortened version of SMAP-29 (1–17, K2,7,13) at the C-terminus (AL-KPP10), which efficiently lysed *Pseudomonas aeruginosa* in the absence of permeabilizers [30]. At the same time, fewer endolysin–peptide combinations have been described to produce effective anti-Gram-negative candidates [2]. More examples are needed to explore the potential of penetration peptides on the bactericidal activity of anti-Gram-negative endolysins.

Previously, we described the LysECD7 endolysin, the putative zinc d-alanyl-d-alanine carboxypeptidase (peptidase M15 family) [23] of the lytic bacteriophage ECD7 (NCBI: txid1981499, *Escherichia coli*), related to the family *Myoviridae* and infecting the *Escherichia* species [31]. The ECD7 phage was isolated from chicken excrement in 2012 using a culture of a Shiga-toxin-producing *E. coli* strain O104:H4, which caused an outbreak of gastroenteritis and hemolytic-uremic syndrome in Germany during 2011 [32]. This phage has lytic activity towards Shiga-like toxin producing *E. coli* strains O104:H4, O157:H7, as well as towards the clinically significant *E. coli* of other serogroups [31]. LysECD7 could be a promising therapeutic agent for antimicrobial drug development, as it showed a wide spectrum of action against Gram-negative bacteria. However, a number of limitations in the use of the LysECD7-8his molecule were revealed; these limitations may interfere with its further development in preclinical and clinical studies [23]. In particular, it was noted that its activity depends on buffer conditions, the presence of salts, and the pH of the medium. Further, the endolysin’s bactericidal effect decreased with the addition of human serum to the assay. These limitations were compensated by the addition of EDTA, which may indicate that the problem was in the membrane permeabilization process under certain conditions [23].

To shed light on the influence of peptide fusions on LysECD7 activity against Gram-negative bacteria, several variants of LysECD7, with different modifications, were designed and produced. Thus, we assessed the effects of biotechnologically relevant histidine tags (6-His, 8-His, and 12-His), as well as a fragment of the permeabilizing peptide SMAP-29, on endolysin’s properties. The effects of the linker between LysECD7 and SMAP-29 were also evaluated. As a result, we found evidence that antimicrobial activity can be significantly modulated by both antimicrobial permeabilizing peptides (e.g., the SMAP-29 fragment peptide) or histidine tags. Taking into account the obtained results, we propose that the tag effect needs to be considered at the early stages of endolysin research and development to mitigate the risks of false conclusions about endolysin’s bactericidal activity.

## 2. Materials and Methods 

### 2.1. Bacterial Strains

The bacterial strains used in the study included laboratory strains and clinical isolates of Gram-negative representatives of the ESKAPE group of pathogens from the collection of the N.F. Gamaleya Federal Research Center for Epidemiology and Microbiology, Ministry of Health of the Russian Federation, from the State collection of pathogenic microorganisms and cell cultures “SCPM-O-B” and from the collection of Gabrichevsky Moscow Research Institute of Epidemiology and Microbiology (Tables S1 and S2). All of the strains were stored at −80 °C and cultivated in an LB broth at 37 °C, at 240 rpm overnight before performing the assays.

### 2.2. Construct Cloning

In this study, synthetic genes were used. LysECD7′s initial coding sequence was artificially synthesized in a pAL-TA commercial vector (Evrogen Ltd., Moscow, Russia), and all endolysin version sequences were derived from it. Briefly, LysECD7 endolysin ORF was amplified from a pALTA-LysECD7 clone (for all primers used, see Table S1) and integrated into the expression vector pET-42b(+) (Evrogen Ltd., Moscow, Russia), resulting in a pET42b-LysECD7-8his plasmid. To obtain pET42b-LysECD7-6his and pET-42b-LysECD7-12his constructs, the whole pET42b-LysECD7-8his vector was amplified with primers 6hisF/6hisR and 12hisF/12hisR, resulting in self-assembly via ligation independent cloning (LIC) expression vectors.

To obtain LysECD7 without tags, the LysECD7 ORF was N-terminally fused to a Mxe intein containing the chitin binding domain (CBD) and cloned into a pBad24 vector (Evrogen Ltd., Moscow, Russia), resulting in pBad24-LysECD7-Mxe plasmid. 

SMAP-fused constructs were also based on a pBAD24 expression vector and contained N-terminal Mxe intein. Three fusions with optimized fragments of the cell membrane permeabilizing antimicrobial peptide SMAP-29 (1–17, K2,7,13, RKLRRLKRKIAHKVKKY) were obtained. They differed in the peptide linker between the LysECD7 endolysin ORF and SMAP-peptide: the pBad24-LysECD7-SMAP-Mxe vector contained no linkers, pBad24-LysECD7-flex-SMAP-Mxe contained a flexible linker (GSAGSAAGSGEF), and pBad24-LysECD7-rigid-SMAP-Mxe contained a rigid linker (AEAAAKEAAAKEAAAKA).

All constructs were checked for errors via Sanger sequencing.

### 2.3. Recombinant Expression and Purification of LysECD7-6his, LysECD7-8his, and LysECD7-12his

Expressed endolysins contained a C-terminal 6-His, 8-His, or 12-His tag for affinity purification. The expression vectors were introduced into the competent *E. coli* cells, strain Rosetta (DE3) (chloramphenicol resistance), using a heat shock transformation protocol. The *E. coli* cells were grown in an LB broth (37 °C, 240 rpm) to an OD600 value of 0.55–0.65 and then induced with β-d-1-thiogalactopyranoside (1 mM IPTG) at 37 °C for 4 h. The cells were harvested by centrifugation (6000× *g* for 20 min at 4 °C) and resuspended in a lysis buffer (20 mM Tris HCl, 250 mM NaCl, and 0.1 mM EDTA, pH 8.0). Then, the suspension was incubated with 100 µg/mL lysozyme at room temperature for 30 min, mixed with 1 mM protease inhibitor PMSF (phenylmethylsulfonyl fluoride), and disrupted by sonication. The cell debris was removed by centrifugation (10000× *g* for 30 min at 4 °C), and the supernatant was filtered through a 0.2-µm filter. The proteins were purified on an NGC Discovery^TM^ 10 FPLC system (Bio-Rad, Hercules, CA, USA) with a 5-mL HisTrap FF column (GE Healthcare, Solingen, Germany) pre-charged with Ni^2+^ ions. The filtered lysate was supplemented with imidazole and MgCl_2_ to a final concentration of 50 mM and 1 mM, respectively, and loaded on a column that was preequilibrated with a binding buffer (20 mM Tris HCl, 250 mM NaCl, and 50 mM imidazole, pH 8.0). The fractions were eluted using a linear gradient to a 100% elution buffer (20 mM Tris HCl, 250 mM NaCl, and 500 mM imidazole pH 8.0). The collected protein fractions were dialyzed against 20 mM Tris HCl pH 7.5. The purity of the proteins was determined by 16% SDS-PAGE (molecular masses are 15.7 kDa for LysECD7-6his, 16.1 kDa for LysECD7-8his and 16.5 kDa for LysECD7-12his, Figure S1). One µg/mL of LysECD7-6his, LysECD7-8his and LysECD7-12his correspond to 0.064, 0.062, and 0.061 µM, respectively. The protein concentrations were measured using a spectrophotometer (Implen NanoPhotometer, IMPLEN, München, Germany) at 280 nm and calculated using a predicted extinction coefficient (1.53, 1.46, 1.45 (mg/mL)^−1^cm^−1^ for LysECD7-6his, LysECD7-8his, and LysECD7-12his, respectively). 

### 2.4. Recombinant Expression and Purification of LysECD7, LysECD7-SMAP, LysECD7-flex-SMAP, and LysECD7-rigid-SMAP

All of the proteins contained a C-terminal intein tag for affinity purification on the Chitin Resin column. The expression vectors were introduced into the competent *E. coli* cells, strain Rosetta (DE3) (chloramphenicol resistance), using a heat shock transformation protocol. The *E. coli* cells were grown in an LB broth (37 °C, 240 rpm) to an OD600 value of 0.55–0.65 and then induced with 0.04% arabinose at 37 °C for 4 h. The cells were harvested by centrifugation (6000× *g* for 20 min at 4 °C) and resuspended in a lysis buffer (20 mM Tris HCl, 250 mM NaCl, 0.1 mM EDTA, and 0.1% Triton X-100 pH 8.0). Then, the suspension was incubated with 100 µg/mL lysozyme at room temperature for 30 min, mixed with 1 mM PMSF, and disrupted by sonication. The cell debris was removed by centrifugation (10,000× *g* for 30 min at 4 °C), and the supernatant was filtered through a 0.2-µm filter. The proteins were purified on an NGC Discovery^TM^ 10 FPLC system (Bio-Rad, Hercules, CA, USA) with a 2-mL column, prepacked with a Chitin Resin matrix (New England Biolabs, Ipswich, MA, USA). The filtered lysate was loaded on a column that was preequilibrated with the binding buffer (20 mM Tris HCl, 250 mM NaCl, 1 mM EDTA, and 0.1% Triton X-100 pH 8.0). Then, the column was washed with the binding buffer and the buffer for intein autosplicing (20 mM Tris HCl, 0.5 M NaCl, 1 mM EDTA, 100 mM DTT pH 8.0) and incubated at room temperature for 4 h at 4 °C overnight. The fractions of the target protein were eluted using an elution buffer (20 mM Tris HCl, 0.5 M NaCl, 1 mM EDTA pH 8.0). The collected protein fractions were dialyzed against 20 mM Tris HCl, 50 mM NaCl, pH 7.5. The purity of the proteins was determined by 16% SDS-PAGE (molecular masses are 14.98 kDa for LysECD7, 17.18 kDa for LysECD7-SMAP, 18.16 kDa for LysECD7-flex-SMAP, and 18.74 kDa for LysECD7-rigid-SMAP, Figure S1). One µg/mL of LysECD7, LysECD7-SMAP, LysECD7-flex-SMAP and LysECD7-rigid-SMAP corresponds to 0.067, 0.058, 0.055, and 0.053 µM, respectively. The protein concentrations were measured using a spectrophotometer (Implen NanoPhotometer, IMPLEN, München, Germany) at 280 nm and calculated using a predicted extinction coefficient (1.57, 1.44, 1.36, 1.32 (mg/mL)^−1^cm^−1^ for LysECD7, LysECD7-SMAP, LysECD7-flex-SMAP, and LysECD7-rigid-SMAP, respectively). 

### 2.5. Antibacterial Assay

Unless otherwise specified, the *Acinetobacter baumannii* Ts 50-16 clinical isolate was used as the strain for the antibacterial activity assays.

Overnight bacterial cultures (OD600 = 1.4–1.6) were used as stationary phase cultures or were diluted 30-fold in an LB broth and grown to the exponential phase (OD600 = 0.6). Subsequently, the cells were harvested by centrifugation (3000× *g*, 10 min) and resuspended in the same volume of phosphate-buffered saline (PBS, pH 7.4). Each suspension was diluted 100-fold in the suitable buffer to a final density of approximately 10^6^ cells/mL. Afterwards, 100 µl of the bacterial suspension and 100 µL of the protein at the appropriate concentrations were mixed in 96-well plates, with a buffer without endolysins used as the negative control. If not otherwise stated, the smallest discriminative concentrations were used to contrast differences in the activities of the LysECD7 fusions. In this case, higher concentrations showed no differences between LysECD7 activities. The mixtures were incubated at 37 °C for 30 min with shaking at 200 rpm and then were diluted 10-fold in PBS (pH 7.4). Subsequently, 100 μL of each dilution was plated onto an LB agar, and the number of surviving bacterial colonies was counted after an overnight incubation at 37 °C. All of the experiments were performed in triplicate, and the antibacterial activity was expressed as follows: Antibacterial activity (%) = 100% – (CFU_exp_/CFU_cont_) × 100%, where CFU_exp_ is the number of bacterial colonies in the experimental culture plates, and CFU_cont_ is the number of bacterial colonies in the control culture plates. Antibacterial activity was regarded as meaningful when it was higher than 33%.

The effects of pH, PBS, blood serum, and growth phase on the bactericidal activity of LysECD7 modifications were analyzed using the *A. baumannii* strain Ts 50-16 cultured to a logarithmic or stationary growth phase. Bacterial suspensions were diluted in 20 mM Tris HCl buffer with different pH values (5.0 to 9.0), PBS pH 7.4, or human blood serum to the required density and mixed with the proteins. Endolysin’s effect on the Gram-negative bacterial strains and clinical isolates was tested using the conditions described above in 20 mM Tris HCl buffer pH 7.5. 

### 2.6. Minimal Inhibitory Concentration Assay

A minimal inhibitory concentration (MIC) assay was performed by the broth microdilution method, as described by Wiegand et al., 2008 [33]. Briefly, an overnight bacterial culture of *Acinetobacter baumannii* Ts 50-16 was grown to the exponential phase (OD600 = 0.6). The cells were harvested by centrifugation and resuspended in phosphate-buffered saline (PBS, pH 7.4). The suspension’s turbidity was adjusted to that of a McFarland Standard 0.5, which is equivalent to 10^8^ CFU/mL, and was then diluted 1:100 in LB broth to 5 × 10^5^ CFU/mL. Subsequently, 50 µL of the bacterial suspension was mixed with 50 µL of the required endolysin’s concentration in 96-well sterile microtiter plates; the final endolysin concentration ranged from 1 µg/mL to 1 mg/mL. Mixtures with LB without endolysin were used as the growth control, and 100 µl of the LB broth alone was used as a sterility control. The microtiter plate was incubated at 37 °C for 16–20 h or until satisfactory growth was obtained. The MIC was defined as the lowest concentration of endolysin that prevented visible growth of the bacteria. All of the experiments were performed in triplicate.

### 2.7. Scanning Electron Microscopy

Overnight *A. baumannii* strain Ts 50-16 bacterial culture was diluted 30-fold in an LB broth and grown to an exponential phase (OD600 = 0.6). Subsequently, 200 µl of the bacterial suspension was spread over a Petri dish with LB agar and left to dry at room temperature. Then, 10 µl of 1 mg/mL LysECD7-8his was dropped onto the bacterial lawn, and the dish was treated for 16 h at 37 °C. Next, the dish was formaldehyde-vapor-fixed with 10% formalin overnight. Imprints of the lysis zone, including the edge and the bacterial lawn, were taken using a silicon plate and were dried for 5 min at room temperature conditions. Next, the samples were mounted to stubs and sputter-coated with a gold layer (5 nm) in an SPI-Module Sputter/Carbon Coater System (SPI Inc., Lakewood, WA, USA). Next, the sputtering samples were analyzed by means of a scanning dual beam electron microscope Quanta 200 3D (FEI Company, Fremont, CA, USA) using the high vacuum mode (10 kV).

## 3. Results

### 3.1. Bactericidal Activity of 8his-Tagged LysECD7

The recombinant LysECD7 was fused to an 8-His tag at the C-terminus (LysECD7-8his) and purified using NiNTA affinity chromatography, as previously characterized by physicochemical and microbiological methods [23]. This molecule showed sufficiently high stability under different conditions and possessed wide bactericidal activity capable of inhibiting the growth of several bacterial species from the ESKAPE group (*Pseudomonas*, *Klebsiella*, *Acinetobacter*, etc). 

**Figure 1:**
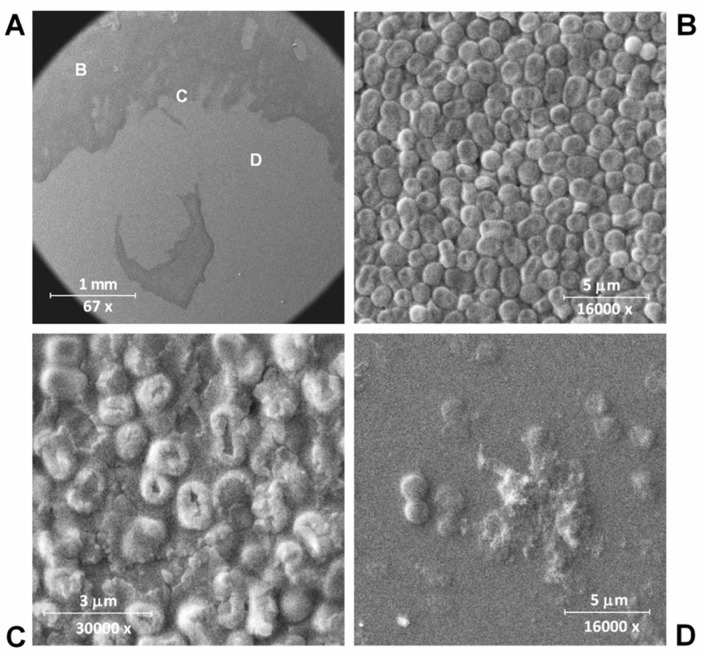
LysECD7-8his-mediated lysis of *A. baumannii* Ts 50-16 cells on plates. Scanning electron microscopy (SEM) images of the plate imprint of the bacterial lysis zone. (**A**) General view of the B, C and D imprints is shown; (**B**) area with no cell lysis (control); (**C**) the edge of the lysis zone; (**D**) the deep lysis zone area. The bacterial lawn was treated for 16 h at 37 °C with 10 μL of LysECD7-8his at 1 mg/mL (10 μg in total) before fixation.

The effect of LysECD7-8his treatment on *A. baumannii* Ts 50-16 clinical isolate cell lysis was visualized using scanning electron microscopy. Scanning electron microscopy (SEM) showed the lytic action of the endolysin against the exponentially growing culture of *A. baumannii* Ts 50-16 (Figure 1). 

After exposure to the LysECD7-8his, the bacterial cells were effectively lysed leaving exposed conglomerates of bacterial cell wall components. The process of cell destruction is clearly visible at the edge of the zone with endolysin (Figure 1C). At the site of the endolysin drop application, almost the complete destruction of *A. baumannii* was observed (Figure 1D). Non-disrupted cells were also observed; however, it is not entirely clear whether they remain viable or not.

**Figure 2:**
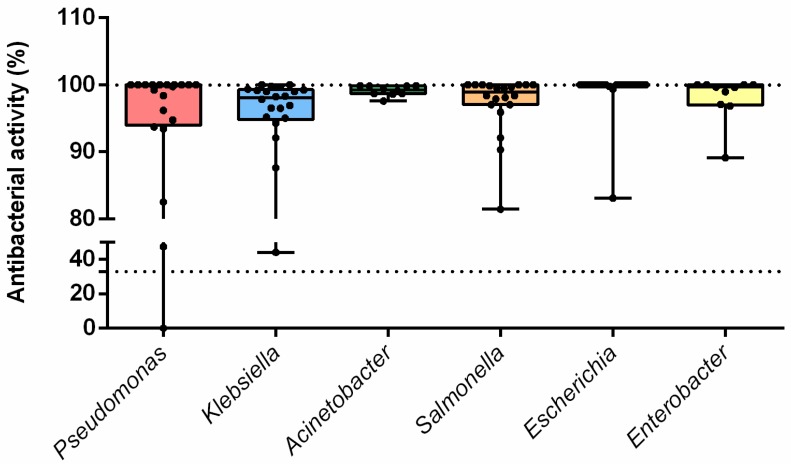
The spectra of antibacterial activity of LysECD7-8his fusion. Lines, medians; boxes, IQR whiskers, min-max. The protein concentration used was 100 µg/mL of LysECD7-8his. The 33% activity cut-off is indicated with a dotted line.

It is known that the host specificity of endolysins can vary significantly, as can their activity against various bacterial genera and species [3]. For most of the experiments in this research, an *A. baumannii* Ts 50-16 clinical isolate was used as a model microorganism, as the LysECD7-8his activity against this strain was previously studied in sufficient detail [23]. However, we also expanded the spectrum of the studied pathogenic microorganisms to more than 100 new strains (Table S2), including *Klebsiella pneumoniae*, *Salmonella* sp., *Pseudomonas aeruginosa*, *Escherichia coli*, *Acinetobacter baumannii*, and *Enterobacter* sp. strains (Figure 2).

LysECD7-8his showed a high antibacterial effect at 100 µg/mL for 101 out of the 102 strains investigated, with antimicrobial activity >33%. The enzyme effectively killed most strains. However, the bactericidal efficiency varied depending on the strain. In line with previous results, endolysin showed wide activity against the studied exponentially grown bacterial species, although resistant strains were also discovered. In particular, no activity against Gram-positive staphylococcal strains was observed. 

### 3.2. Influence of the Length of Polyhistidine Tag on Antibacterial Properties of LysECD7

**Figure 3:**
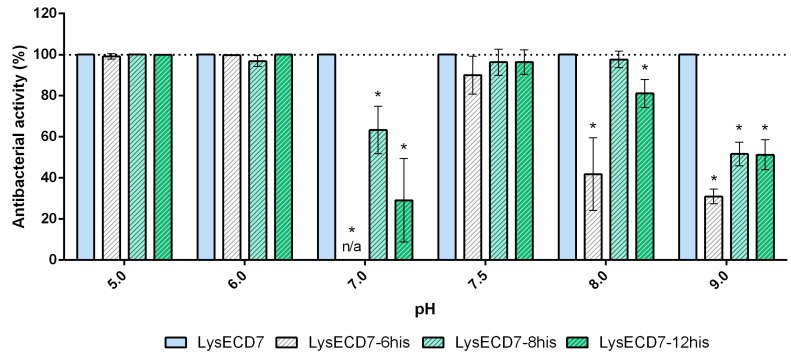
The effect of the histidine tag on the lytic properties of LysECD7 against the *A. baumannii* Ts 50-16 strain over a pH gradient range at a protein concentration of 1 µg/mL. The bacterial suspension was diluted in 20 mM Tris HCl buffer with different pH values (5.0–9.0) to 1 to 3 × 10^5^ CFU/mL and mixed with the proteins. For all experiments, the mean values are shown from three independent experiments (± standard deviation, SD). n/a, no bactericidal activity detected. An asterisk (*) indicates a significant difference in bactericidal activity compared to the LysECD7 enzyme (*p* < 0.05, two-way ANOVA with Dunnett’s multiple comparisons test).

Previously, it was suggested that a polyhistidine tag can serve as an unspecific permeabilizing peptide, affecting the putative translocating activity of endolysins and expanding the spectrum of their action [23,34]. To assess the influence of the histidine tag on bactericidal activity, four different LysECD7 constructions were compared: a 6-His residue fusion (LysECD7-6his), an 8-His residue fusion (LysECD7-8his), and a 12-His residue fusion (LysECD7-12his) (Figure 3), as well as an LysECD7 molecule without a tag to exclude the effect of the histidine tag.

To contrast the differences in the permeabilization activity of LysECD7 variants on *A. baumannii* Ts 50-16 over a pH gradient, a concentration of 1 µg/mL was used (0.061–0.067 µM for different modifications). Analysis of the activity over a range of pH on the model of the *A. baumannii* strain showed that at acidic pH values, maximal activity was observed, regardless of the molecule used. Further, with an increase in pH, fluctuations of activities can be noted. LysECD7 was active at a concentration of 1 µg/mL over the whole pH spectra. Among all tagged variants, LysECD7-6his activity was the least stable, with a significant loss of activity at pH 7.0, 8.0, and 9.0. LysECD7-8his and LysECD7-12his (Figure 3) also lost about half of their activity at pH 7.0 and 9.0. In general, all His-tagged enzymes were less active then the native LysECD7. LysECD7-8his and LysECD7-12his had close activity profiles and were more active in the pH range than LysECD7-6his. At the same time, the addition of four histidine residues of LysECD7-12his fusion compared to 8-His construction did not have a beneficial effect on bactericidal activity. For pH 7.0 and pH 8.0, the activity of LysECD7-12his was significantly less than that of LysECD7-8his. Despite similar activity profiles, the molecules differed significantly depending on the tag length added to the native protein sequence. LysECD7-8his was the most effective in a broad pH range among the His-tagged molecules.

### 3.3. Improvement of Endolysin LysECD7 Bactericidal Activity through a Fragment of SMAP-29 Peptide Fusion

To obtain endolysins capable of overcoming the protective outer membrane with a lipopolysaccharide layer of Gram-negative bacteria, different approaches were investigated. One of these approaches involved the construction of engineered endolysins fused to either the N- or the C-terminus of membrane-destabilizing peptides. 

Here, we assessed the influence of the antimicrobial peptide SMAP-29 fragment fused to the C-terminus of LysECD7 on the bactericidal activity of the enzyme. Three modified variants of LysECD7 were produced, including LysECD7-SMAP and two additional modifications, LysECD7-flex-SMAP and LysECD7-rigid-SMAP, carrying a Gly-Ser-rich linker or an alanine-rich helical linker between endolysin and the antibacterial peptide, SMAP-29 [35]. An optimized fragment of the SMAP-29 (1–17, K2,7,13, RKLRRLKRKIAHKVKKY) sequence with improved antimicrobial activity and without hemolytic toxicity was used [28,29]. 

The activity of the modified endolysins was assessed under the spectra of different conditions, including those in which the 8-His-tagged variant of LysECD7 showed decreased activity or did not act at all according to previous data [23]. The LysECD7 activity in the presence of PBS buffer and serum, as well as its activity on bacteria in different growth phases was evaluated. 

**Figure 4:**
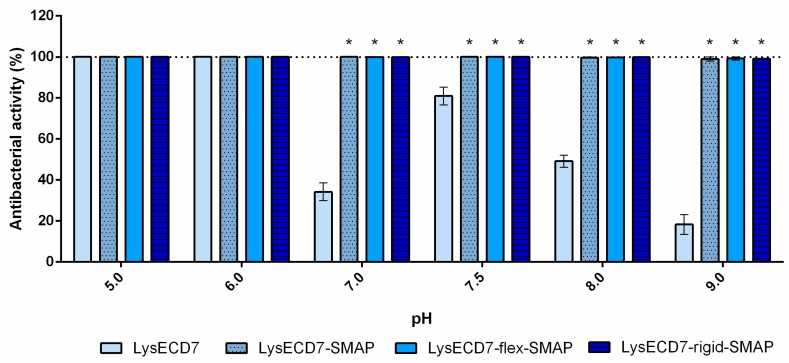
Bactericidal activity of SMAP-modified LysECD7 against the *A. baumannii* Ts 50-16 strain over the pH gradient range at a protein concentration of 0.5 µg/mL. The bacterial suspension was diluted in 20 mM Tris HCl buffer with different pH values (5.0 to 9.0) to 1 to 3 × 10^5^ CFU/mL and mixed with the proteins. For all experiments, the mean values are shown from three independent experiments ± SD. An asterisk (*) indicates a significant difference in bactericidal activity compared to the LysECD7 enzyme (*p* < 0.05, two-way ANOVA with Dunnett’s multiple comparisons test).

Figure 4 shows the bactericidal activity of endolysins over a pH gradient range at the protein concentration of 0.5 µg/mL (0.027–0.034 µM). No deviations in the neutral pH, characteristic for His-tagged variants, were observed compared to the LysECD7 enzyme. All SMAP-modified molecules retained 100% activity over the entire pH range.

Thus, apparently, the limitations of LysECD7 histidine-tagged variants are associated with the penetration of the cell membrane and the availability of the peptidoglycan substrate. 

**Figure 5:**
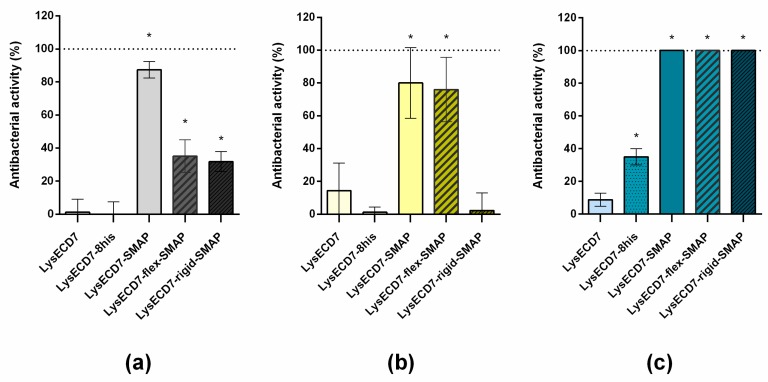
Bactericidal activity of the modified endolysins. (**A**) Activity of the endolysins against *A. baumannii* Ts 50-16, diluted in a PBS buffer solution (protein concentration of 1 µg/mL); (**B**) activity of endolysins against *A. baumannii* Ts 50-16, diluted in human serum (protein concentration of 10 µg/mL); (**C**) activity of lysins against the stationary-phase cells of *A. baumannii* Ts 50-16, diluted in 20 mM Tris HCl buffer pH 7.5 (protein concentration of 10 µg/mL). Culture dilution was 1 to 3 × 10^5^ CFU/mL. For all experiments, the mean values are shown from three independent experiments ± SD. An asterisk (*) indicates a significant effect on bactericidal activity compared to the LysECD7 enzyme (*p* < 0.05, one-way ANOVA, Dunnett’s multiple comparisons test).

We also compared the bactericidal activity of SMAP-modified endolysins in the presence of PBS buffer against the stationary phase growth of *A. baumannii* 50-16 and in blood serum (Figure 5).

The significant problem of LysECD7-8his for subsequent practical use is a bactericidal activity drop in PBS (Figure 5A) and blood serum (Figure 5B). PBS simulates the physiological conditions of acidity and salinity and is widely used with injection drugs, while blood serum is the most complex medium that is closest to in vivo conditions. At the same time, all SMAP-modified molecules exert superior activity under these conditions and at the bacterial stationary phase (Figure 5, Figure S2). 

The assessment of the activity of all of molecules in PBS demonstrated the lower bactericidal activity of fusions, with both linkers at a concentration of 1 µg/mL (0.053–0.067 µM)—35% for LysECD7-flex-SMAP and 31% for LysECD7-rigid-SMAP, compared to LysECD7-SMAP, which retained activity up to 87% (Figure 5A). At a concentration of 10 µg/mL (0.53–0.67 µM), there were no differences between the three SMAP-modified endolysins with 100% bactericidal activity, while native LysECD7 possessed only 13% bactericidal activity (Figure S2, Panel A). The differences in dose dependency as well as drop in proteins activity can be explained by the effect of diluent used, as in Tris buffer the cells will have a higher internal turgor pressure compared to those resuspended in PBS, promoting their lysis.

In human blood serum, LysECD7-flex-SMAP and LysECD7-SMAP without linkers showed the best activity, with 76% and 80%, respectively (Figure 5b). The LysECD7-rigid-SMAP protein almost completely lost its activity and acted similar to or worse than LysECD7-8his and LysECD7. Thus, LysECD7-SMAP and LysECD7-flex-SMAP were less affected by the conditions of reactions among the investigated molecules.

Moreover, SMAP-modified enzymes were active against stationary phase grown bacteria, compared with LysECD7 (Figure 5c and Figure S2, Panel B). 

According to the results described above, among His-fusions the most active molecule is LysECD7-8his and for SMAP-fusions is LysECD7-SMAP. We compared the MIC values for these two molecules and LysECD7 without tags. The visible growth of the *Acinetobacter baumannii* Ts 50-16 strain was inhibited by 50 µg/mL of LysECD7-SMAP, while for LysECD7 and LysECD7-8his, MIC was not established, as it was >1 mg/mL.

### 3.4. Assessment of the Bactericidal Activity of SMAP-Modified Endolysin against LysECD7-8his Recalcitrant Strains

Modifications of lytic enzymes with permeabilizing peptides are primarily aimed to increase endolysin activity, as well as to expand the spectrum of bacterial strains sensitive to endolysin’s action. Modified endolysins are engineered to obtain high bactericidal activity against Gram-negative bacteria [24]. Among the studied bacteria (see Section 3.1, Table S3), several strains also showed low or no sensitivity towards LysECD7-8his. Here, we compared the ability of modified endolysins with His- and SMAP-tags to act against these strains of Gram-negative bacteria. We also estimated the effects on two Gram-positive bacterial strains (*Staphylococcus aureus* and *S. haemolyticus*). 

**Figure 6:**
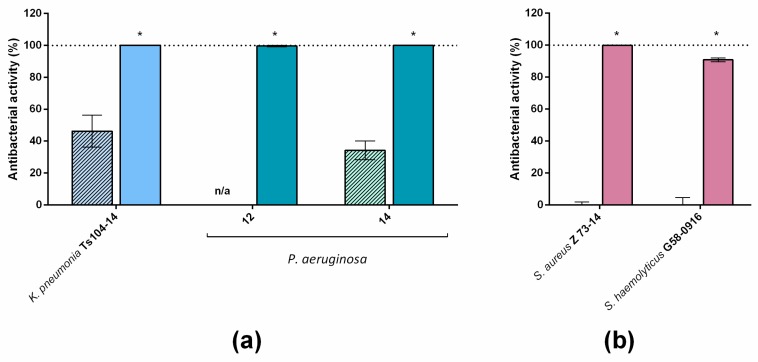
Bactericidal activity of LysECD7-8his (hatched bars) and LysECD7-SMAP (empty bars) on the recalcitrant strains of Gram-negative (**a**) and Gram-positive (**b**) bacterial isolates diluted in 20 mM Tris HCl buffer pH 7.5. All proteins were assessed at a concentration of 50.0 µg/mL and the culture dilution was 1 to 3 × 10^5^ CFU/mL. For all experiments, the mean values are shown from three independent experiments ± SD. n/a, no bactericidal activity detected. An asterisk (*) indicates a significant effect on bactericidal activity compared to the LysECD7-8his enzyme (*p* < 0.05, Mann–Whitney test).

The visible effect for LysECD7-8his was observed only for a concentration of 50.0 µg/mL (3.1 µM), while LysECD7-SMAP at the same concentration (2.9 µM) completely inhibited the growth of all the strains (Figure 6). Also, at this concentration, LysECD7-SMAP was active against two staphylococcal isolates. In general, a concentration of 1 µg/mL for the most active of the investigated SMAP-variants (LysECD7-SMAP) was enough to eliminate the growth of exponentially grown bacterial cells of the *K. pneumoniae* recalcitrant strain in vitro.

The contribution of SMAP peptide into the LysECD7-SMAP antibacterial activity was assessed using the SMAP fusion with the AmilCP chromogenic protein, which does not possess bactericidal activity itself (Figure S3). The additive effect of SMAP and LysECD7 parts was revealed. Bactericidal activity of AmilCP-SMAP fusion was relatively high (50–95%) against *A. baumannii* and *K. pneumoniae* and low (6–8 %) against *S. aureus* and *S. haemolyticus*. Thus, for Gram-positive bacterial strains the synergistic effect was much stronger than for Gram-negative, where LysECD7 without tags was able to act independently.

## 4. Discussion

Endolysins, as well as their engineered derivatives, offer great opportunities for the development of antimicrobials in the face of the growing problem of antibiotic resistance of pathogenic microorganisms. In vitro experiments demonstrated that endolysins exert bactericidal activity against Gram-negative strains in concentrations varying from 1 to 500 µg/mL (0.06–6.2 µM) [18,20,23,34] and values significantly depended on the strains used. For LysECD7-8his, it was shown that less than 100 μg/mL (6.2 µM) was enough to eradicate growing bacteria up to five orders of magnitude. 

However, it is difficult to predict the enzyme’s therapeutic potential in vivo in advance, as complex internal body environments are often not reflected by model conditions. For example, it was shown that 200 µg of P307SQ-8C peptide (endolysin PlyF307 derivative) reduced the bacterial load up to 2 logs in an *A. baumannii* mouse skin infection model when used locally [36], while in the mouse *Acinetobacter* sepsis model, 1 mg of subcutaneously applied PlyF307 significantly increased the rate of survival [20]. Thus, it is difficult to precisely approximate the in vivo therapeutic potential of endolysins based solely on in vitro data. 

Diverse peptide “add-ons” help to modify the activity of lytic enzymes. To shed light on the influence of C-terminal peptide tags on endolysin activity against Gram-negative bacteria, LysECD7 was chosen as the model enzyme. Here, we demonstrated how the properties of enzymes can be changed after histidine tag or SMAP fusions.

It was assumed that the exogenous effect of endolysins on Gram-negative bacteria is associated with the presence of a highly positively charged C-terminal amphipathic helix in the molecule, with a likely role in promoting outer membrane penetration [36,37]. Despite the absence of this predicted positively charged stretch of amino acids in LysECD7-8his, it was effective against various bacterial strains of *Pseudomonas aeruginosa, Acinetobacter baumannii, Klebsiella pneumoniae, Escherichia coli*, and *Salmonella typhi*. It was suggested that membrane penetration occurs non-specifically through positively charged His-tag clusters (KLEHHHHHHHH). This hypothesis was supported by the correlation of charge and the bactericidal activity of LysECD7-8his under different pH values (Spearman’s rank correlation coefficients *r* = 0.73) [23]. Here, we investigated the process in more detail. For this investigation, three constructions with different histidine tag lengths (6, 8, and 12 residues) were compared with LysECD7 activity and without His-tag in the pH range. In the acidic pH range, there were no differences between the modified enzymes, which is typical for endolysins, which work perfectly under acidic conditions [34]. Further differences were revealed in the spectra of action for the enzymes, with 6-His being significantly less active than 8- and 12-His molecules. However, all His-tagged enzymes significantly lost their activity compared to the native enzyme.

The effect of the histidine tag was studied previously. For example, for recombinant His-tagged phage metallopeptidase, EndoT5 purification on the Ni-Sepharose carrier led to a significant drop in enzymatic activity [38]. However, a detailed study showed that the purification method affected the activity rather than the tag itself. Additional histidine residues alone did not significantly affect the folding of the enzyme or its structural properties. The addition of the His-Tag caused a noticeable reduction in the protein’s conformational stability and resulted in a dramatic decrease in shelf-life due to the presence of imidazole and nickel ions accompanying the purification of a protein by IMAC. 

The results described in this paper demonstrate that shortening the histidine tail significantly decreased the activity of LysECD7-6his, while the bactericidal effect of the LysECD7-12his and LysECD7-8his was relatively high in a broad pH spectrum. We previously proposed that these dynamics could be explained by the additional protonation of C-terminal cationic peptides of endolysins and their increased destabilizing efficiency towards the bacterial outer membrane under a low pH range (pH 5.0 and 6.0), presumably adjusted with the 8-histidine tag sequence with estimated charge (pKa) ranging from 8.00 to −1.00 at pH 5 to 9 [23]. We showed that a decrease in activity is primarily due to the enzymes’ endogenous ability to penetrate the bacterial membrane, since in the presence of EDTA, bactericidal activity recovered. However, in the case of LysECD7, this correlation was not as evident, as it had no predicted structural elements in its secondary and tertiary structures that could condition this effect. Moreover, the activity recovery at pH 8.0 and 9.0 did not correlate with the charges of either the C-termini or the complete LysECD7 protein sequence. The retained level of activity of LysECD7 without modifications in a concentration of 1 µg/mL (0.067 µM) (Figure 3) under the whole pH spectrum suggests that this problem can be partly associated with the introduction of His-tags or the method of purification of such enzymes. On the other hand, in smaller concentrations (0.5 µg/mL/0.034 µM of LysECD7, Figure 4), we observed a characteristic activity reduction, suggesting that this process concerns more than just a protein production method. We assume the presence of additional functional elements in the protein structure that are capable of changing the non-specific permeabilization of the outer cell membrane under certain media conditions. To date, it remains difficult to localize this structure in the protein sequence; however, structural studies of the LysECD7 protein and its variants could shed light on this issue.

This means that histidine tagging definitely affects the bactericidal properties of endolysin, but the charge hypothesis does not completely explain these alterations, and the particular effects of different lengths of fusions are difficult to predict. Most of the endolysins described today are 6-His fusions [2,6,16,17,18,19,20,21,22]. How the activity of all these endolysins are modified by 6-His tag fusion has not yet been described in detail. In light of our study, 6-His fusion LysECD7-6his demonstrates the worst bactericidal properties compared to 8- and 12-His fusions. This result indicates that a number of histidines at the C-terminal significantly affect bactericidal activity and should be selected experimentally to exclude the possibility of wrong conclusions about lysin bactericidal activity.

A study of the effects of LysECD7-8his on an extended spectrum of bacterial strains showed that the enzyme exerts bactericidal activity on 101 out of 102 strains. However, several isolates were resistant to LysECD7-8his in different species (Figure 1). In terms of the number of strains tested, this study of the bactericidal action of endolysin is among the largest studies currently published [24,39]. The reason for the wide mode of action of endolysins remains unknown. It is assumed that the positively charged N- or C-terminal domain can interfere with the negatively charged LPS layer, thus allowing endolysins to penetrate through the bacterial outer membrane [16,17]. However, the emergence of an increasing amount of indirect evidence [16,34,36,40] proposes also the complex structure for relatively small endolysins from Gram-negative targeting phages. Thus, the outer membrane permeabilizing activity was previously shown for two highly cationic C-terminal peptides P307 [36] and LysAB2 P3 [16] of phage endolysins. Based on these data, it can be speculated that the enzyme’s activity is manifested not only due to lysin’s catalytic activity but also due to nonspecific destruction of the bacterial outer membranes. Unfortunately, there is a lack of direct studies available confirming this hypothesis.

The construction of genetically modified endolysins with altered properties allows us to increase the effectiveness of enzymes, especially with respect to Gram-negative pathogens. For this, the effects of various antimicrobial peptides with cell membrane permeabilization effects have been actively studied, among which the sheep myeloid antimicrobial peptide SMAP-29 occupies an important place. A large number of SMAP-29 variants are described in the literature, differing in their lengths and modifications [27]. This is due to the fact that the native polypeptide itself possesses a high cytotoxicity and hemolytic effect, so active but less toxic variants are needed.

Endolysin fusions with SMAP-29 have already been investigated. An optimized homolog of KZ144 endolysin with SMAP-29 peptide, which was considered to be a highly potent antibacterial that kills virtually all *P. aeruginosa* strains, acts quickly, is recalcitrant to resistance development, and is able to kill metabolically inactive persisters [6]. The production of such chimeras of endolysins can be associated with certain difficulties in expression, presumably because these proteins can be highly toxic to the producer strain [34]. In this case, the proteins may be expressed by the cells in an insoluble fraction or not expressed at all. The introduction of the linker between the domains can help in this situation, but its correct selection is particularly important for the construction of functional fusion proteins [41,42,43].

Here, we evaluated the effects of three different chimeras, including the fusion between LysECD7 and optimized SMAP-29 through a Gly- and Ser-rich flexible linker (flexible, GSAGSAAGSGEF) [44], which avoided large hydrophobic residues to maintain good solubility in aqueous solutions, and a monomeric α-helix linker (rigid, AEAAAKEAAAKEAAAKA) [45], which is frequently used when it is crucial to keep a fixed distance between the functional domains for their spatial separation to maintain the stability or bioactivity of the fusion proteins [46]. Among the three fusions, the LysECD7-SMAP without additional linkers appeared to be the most effective, facilitating retrieval of the active protein in conditions inconvenient for the native enzyme or its His-tagged forms. The two linkers also increased the activity of the LysECD7 enzyme but in a less effective manner.

The obtained data propose that the bactericidal activity of LysECD7 and its derivatives is highly dependent on environmental conditions. Thus, the endolysin’s bactericidal activity is weak under conditions supporting bacterial growth, as MIC values determined in LB medium are high (50 µg/mL and more than 1 mg/mL). At the same time, significant bactericidal activity was observed when target cells were assayed in PBS or Tris buffer solutions.

The introduction of the optimized SMAP-29 peptide into the endolysin sequence with different variants expanded the spectrum of enzyme activity in vitro, allowing us to kill even recalcitrant strains not previously affected by LysECD7-8his and to almost completely eliminate the viable bacteria in the presence of complex media, such as blood serum. Moreover, the activity of C-terminal SMAP peptide fused with a protein lack of antibacterial activity (AmilCP chromogenic protein) showed different effectiveness, compared to LysECD7 and LysECD7-SMAP (Figure S3). Although the resulting hybrid AmilCP-SMAP was able to reduce the bacterial CFU, especially in the case of Gram-negative bacteria, such reduction was lower compared to LysECD7-SMAP, declaring the synergic effect of endolysin–peptide hybridization. The permeabilizing activity of the peptide does not explain the acquired ability of the molecules to act against Gram-positive bacteria. There is evidence that antimicrobial peptides can perform not only the role of a permeabilizer but also selectively bind to bacterial membranes using LPS or phospholipid monolayers containing negatively charged lipids as the initial binding site in the killing process of bacteria [47,48,49,50]. However, data on this aspect of antimicrobial peptides are controversial. Tack et al. [51] reported that LPS binding was found to correlate with antimicrobial potency, while Bartlett et al. [47] found that the LPS binding of SMAP-29 and shorter analogues was not correlated with antimicrobial activity toward two Gram-negative bacteria (*P. aeruginosa* and *K. pneumoniae*). In this work, we did not separate the endolysin’s catalytic action on peptidoglycan substrate and SMAP membrane permeabilizing action from each other. Thus, the contribution of both LysECD7 catalytic domain with predicted endopeptidase activity and antimicrobial peptide sequence to the bactericidal activity of LysECD7-SMAP fusion, as well as its interactions with the substrate, is to be clarified. In this relation structural analysis followed by the site-directed mutagenesis is substantial, and study of the dynamics of the protein secondary structure is of vital importance.

## 5. Conclusions

The modular nature of bacteriophage endolysins provides a great opportunity for the engineering construction of these enzymes to modulate their activity. In this paper, we showed that the introduction of various biotechnological tags can dramatically affect the activity of the enzyme.

In particular, we obtained SMAP-modified versions of LysECD7, largely devoid of the disadvantages of the native enzyme, with high bactericidal activity over the pH range, resistance to the inhibiting action of salt in PBS and human blood sera, and activity in vitro, not only against Gram-negative, but also against Gram-positive, bacteria. The molecules’ high activity against the stationary phase cells indicates a potentially high bactericidal effect against the dormant bacterial cells forming biofilms. All these properties are good evidence that, based on these molecules, it will be possible to develop antimicrobials that can be highly active not only during topical treatment but also for systemic applications in the bloodstream and tissues. This opens up the possibility of developing more effective drugs based on SMAP-modified LysECD7, with stable and predictable properties against different pathogens.

## Supplementary Materials

The following are available online at https://www.mdpi.com/2218-273X/10/3/440/s1: Table S1: Primers used in the study; Table S2: Bacterial strains used to determine the spectrum of activity of LysECD7-8his and the antibacterial activity of this protein at a protein concentration of 100 µg/mL; Table S3: Bacterial strains used to compare the bactericidal activity of LysECD7-8his and LysECD7-SMAP and the antibacterial activity of these proteins at protein concentrations 50 µg/mL; Figure S1: Schematic representation of expression constructs used in this study; Figure S2: Efficiency of modified endolysins LysECD7 under different conditions.
