# Supplementary material for: Modulation of Endolysin LysECD7 Bactericidal Activity by Different Peptide Tag Fusion
Source: Biomolecules. 2020 Mar 12;10(3):440. doi: 10.3390/biom10030440 (PMC7175214; doi:10.3390/biom10030440)
Supplement: Supplementary file 1 [file biomolecules-10-00440-s001.pdf]

Modulation of Endolysin LysECD7 Bactericidal Activity by Different Peptide Tag Fusion

Nataliia P. Antonova [1,2], Daria V. Vasina [1,3], Evgeny O. Rubalsky [4,8,9], Mikhail V. Fursov [5], Alina S. Savinova [1], Igor V. Grigoriev [1], Evgeny V. Usachev [1], Natalia V. Shevlyagina [1], Vladimir G. Zhukhovitsky [1,7], Vadim U. Balabanyan [2], Vasiliy D. Potapov [5], Andrey V. Aleshkin [4], Valentine V. Makarov [6], Sergey M. Yudin [6], Alexander L. Gintsburg [1,7], Artem P. Tkachuk [1] and Vladimir A. Gushchin [1,2,*]

Supplementary materials

Table S1. Primers, used in the study.

	5'-PrimerSequence-3′	
pET42vecF	5′-CTCGAGCACCACCACCACCACCACCACCACTAA-3′	Vector part
pET42vecR	5′-CATATGTATATCTCCTTCTTAAAGTTAAACAAAAT-3′	
ECD7F	5′-AAGAAGGAGATATACATATGTTTAAATTATCTCAAAGAAGCA-3′	LysECD7 ORF
ECD7R	5′-TGGTGGTGGTGGTGCTCGAGTTTTGGATCTGGGTATTTGCT-3′	
	LysECD7-Polyhistidine constructions	
6HisF	5′-AAACTCGAGCACCACCACCACCACCACTAATTGATTAATACCTAGGCTGCTAAAC-3′	LysECD7-6His
6HisR	5′-AATCAATTAGTGGTGGTGGTGGTGGTGCTCGAGTTTTGGATCTGGGTAT-3′	
12HisF	5′-AAACTCGAGCACCACCACCACCACCACCACCACCACCACCACCACTAATTGATTAATACCTAGGCTGCTAAAC-3′	LysECD7-12His
12HisR	5′-AATCAATTAGTGGTGGTGGTGGTGGTGGTGGTGGTGGTGGTGGTGCTCGAGTTTTGGATCTGGGTAT-3′	
	LysECD7-Intein constructions	
MxeVecF	5′-TGCATCACGGGAGATGCACT-3′	ECD7 sequence fused with Mxe containing vector
pB24Rvec	5′-CATATGTATATCTCCTTCTTAAAGTTAAACAAAATTATTTC-3′	
ECD7MXER	5′-AGTGCATCTCCCGTGATGCAAGCCTCGAGTTTTGGATCTGGGTATTTGCT-3′	
	LysECD7-SMAP constructions	
Ecd7SmapMxeR	5′-AGTGCATCTCCCGTGATGCAATACTTCTTCACCTTATGTGCTATCTTCCTCTTCAGTCTTCGAAGCTTCCTCTCG AGTTTTGGATCTGGGTATTTGCT-3′	LysECD7-SMAP
RigF	5′-aggaggctgcggcaaaagaagcggctgccaaggcaAGGAAGCTTCGAAGACTGAAGAGG-3′	rigid linker
ECD7RigR	5′-ttcttttgccgcagcctccttggcagccgcctctgcCTCGAGTTTTGGATCTGGGTATTTGCT-3′	
FlexF	5′-ctgggtctgcagcaggttcgggcgagttcAGGAAGCTTCGAAGACTGAAGAGG-3′	flexible linker
ECD7FlexR	5′-cgaacctgctgcagacccagccgagccCTCGAGTTTTGGATCTGGGTATTTGCT-3′	
	AmilCP-SMAP construction	
SmapF	5′-AGGAAGCTTCGAAGACTGAAGAGGAAG-3′	Vector part
pB24Rvec	5′-CATATGTATATCTCCTTCTTAAAGTTAAACAAAATTATTTC-3′	
pB24-AmilCP	5′-TTTGTTTAACTTTAAGAAGGAGATATACATatgagtgtgatcgctaaacaaatgacct-3′	AmilCP-SMAP
AmilCP-SMAPR	5′-CTTCAGTCTTCGAAGCTTCCTGGCGACCACAGGTTTGCGTGCA-3′	

Table S2. Bacterial strains used to determine the Spectrum of activity of LysECD7-8his. Antibacterial activity* of this protein and CFU reduction* at concentration of protein upon treatment of 10^3 to 10^5 CFU/ml with 100 μg/ml.

**Strain**	**Source**	**Method of Identification**	**Antibacterial Activity**	**CFU Reduction, CFU/ml**
Salmonella entérica subsp. *enterica* serovar Enteritidis «Kaluga»	Animal infectious disease, chicken		97.92%	4.95 x 10^4
Salmonella enterica subsp. *enterica* serovar Infantis 4632	Animal infectious disease, chicken		100.00%	6.45 x 10^4
Salmonella enterica subsp. *enterica* serovar Tiphimurium «Lo»	Animal infectious disease, pigeons		100.00%	2.27 x 10^4
Salmonella enterica subsp. *enterica* serovar Enteritidis 3B	Animal infectious disease, chicken		100.00%	6.55 x 10^4
Salmonella enterica subsp. *enterica* serovar Infantis 2511	Animal infectious disease, chicken		100.00%	8.60 x 10^4
Salmonella enterica subsp. *enterica* serovar Tiphimurium 415	Animal infectious disease, chicken		100.00%	3.50 x 10^3
Salmonella enterica subsp. *enterica* serovar Tiphimurium 1281	Animal infectious disease, chicken		99.75%	8.08 x 10^4
Salmonella enterica subsp. *enterica* serovar Tiphimurium Ufa	Animal infectious disease, ducks		100.00%	4.25 x 10^4
Salmonella enterica subsp. *enterica* serovar Tiphimurium #3	Animal infectious disease, ducks	Cultural, morphological and biochemical properties, serological typing	97.04%	5.58 x 10^4
Salmonella enterica subsp. *enterica* serovar Tiphimurium #24	Animal infectious disease, chicken		92.07%	6.45 x 10^4
Salmonella enterica subsp. *enterica* serovar Enteritidis 25	Animal infectious disease, chicken		98.43%	3.98 x 10^4
Salmonella enterica subsp. *enterica* serovar Infantis 4631	Animal infectious disease, chicken		100.00%	1.19 x 10^5
Salmonella enterica subsp. *enterica* serovar Infantis 4522	Animal infectious disease, chicken		100.00%	6.30 x 10^4
Salmonella enterica subsp. *enterica* serovar Dublin immobilin 2	Animal infectious disease, calf		99.88%	8.19 x 10^4
Salmonella enterica subsp. *enterica* serovar Enteritidis «4B»	Animal infectious disease, chicken		99.50%	3.98 x 10^4
Salmonella enterica subsp. *enterica* serovar Infantis «Kuzn»	Animal infectious disease, chicken		100.00%	9.15 x 10^4
Salmonella enterica subsp. *enterica* serovar London N2	Animal infectious disease, turkey		71.30%	7.95 x 10^4
Salmonella enterica subsp. *enterica* serovar Tiphimurium G1	Livestock wastes	Cultural, morphological and biochemical properties, MALDI-TOF	99.49%	1.55 x 10^5
Salmonella	Livestock	Cultural, morphological and	82.62%	2.52 x 10^4

enterica subsp. *enterica* serovar Enteritidis SEM4	wastes	biochemical properties		
Salmonella enterica subsp. *enterica* serovar Infantis 1271	Livestock wastes	Cultural, morphological and biochemical properties, MALDI-TOF	91.52%	1.54 x 10^{4}
Pseudomonas aeruginosa 1	Hospital strain, inpatient hospital	Cultural, morphological and biochemical properties, MALDI-TOF	100.00%	2.50 x 10^{4}
Pseudomonas aeruginosa 2			100.00%	9.30 x 10^{3}
Pseudomonas aeruginosa 3			100.00%	1.53 x 10^{4}
Pseudomonas aeruginosa 4			100.00%	5.00 x 10^{2}
Pseudomonas aeruginosa 5			100.00%	3.70 x 10^{3}
Pseudomonas aeruginosa 6			100.00%	1.05 x 10^{3}
Pseudomonas aeruginosa 7			100.00%	6.35 x 10^{4}
Pseudomonas aeruginosa 8			100.00%	1.66 x 10^{4}
Pseudomonas aeruginosa 9			100.00%	1.13 x 10^{4}
Pseudomonas aeruginosa 10			100.00%	7.75 x 10^{3}
Pseudomonas aeruginosa 11			81.90%	1.43 x 10^{5}
Pseudomonas aeruginosa 12			0%	0
Pseudomonas aeruginosa 13			93.75%	3.00 x 10^{5}
Pseudomonas aeruginosa 14			47.79%	3.25 x 10^{3}
Pseudomonas aeruginosa 15			98.37%	1.48 x 10^{5}
Pseudomonas aeruginosa 16			96.19%	1.24 x 10^{5}
Pseudomonas aeruginosa 3086	Reference laboratory strain	Cultural, morphological and biochemical properties, MALDI-TOF	99.75%	4.67 x 10^{5}
Pseudomonas aeruginosa 1805	Hospital strain, inpatient hospital		99.46%	5.57 x 10^{4}
Pseudomonas aeruginosa PA01	Reference laboratory strain		94.48%	5.95 x 10^{5}
Pseudomonas aeruginosa B-1304	Reference laboratory strain	Cultural, morphological and biochemical properties, MALDI-TOF	99.26%	3.77 x 10^{5}
Klebsiella pneumoniae 1	Hospital strain, inpatient hospital	Cultural, morphological and biochemical properties	97.85%	5.24 x 10^{4}
Klebsiella pneumoniae 2			98.22%	4.42 x 10^{4}
Klebsiella pneumoniae 3			95.16%	5.90 x 10^{4}
Klebsiella pneumoniae 4			96.50%	5.94 x 10^{4}
Klebsiella pneumoniae 5			96.52%	8.74 x 10^{4}
Klebsiella pneumoniae 6			99.79%	9.63 x 10^{4}
Klebsiella pneumoniae 7			99.03%	5.60 x 10^{4}
Klebsiella pneumoniae 8			99.38%	9.64 x 10^{4}
Klebsiella pneumoniae 9			99.18%	9.67 x 10^{4}
Klebsiella pneumoniae 10			87.62%	6.44 x 10^{4}
Klebsiella pneumoniae 11			94.29%	3.63 x 10^{4}
Klebsiella pneumoniae 12			95.03%	8.70 x 10^{4}
Klebsiella pneumoniae 13			100.00%	1.96 x 10^{4}
Klebsiella pneumoniae 14			99.22%	3.18E x 10^{4}
Klebsiella pneumoniae 15			98.31%	9.59 x 10^{4}
Klebsiella pneumoniae 16			99.00%	5.94 x 10^{4}
Klebsiella pneumoniae 17			100.00%	9.75 x 10^{4}
Klebsiella pneumoniae 18			96.95%	9.21 x 10^{4}
Klebsiella pneumoniae 19			99.73%	9.18 x 10^{4}
Klebsiella pneumoniae 20			92.16%	8.53 x 10^{4}
Klebsiella pneumoniae Ts 104-14	Hospital strain, outpatient hospital	Cultural, morphological and biochemical properties, MALDI-TOF	72.65%	7.61 x 10^{3}
Escherichia coli 96	Hospital strain, inpatient hospital	Cultural, morphological and biochemical properties, MALDI-TOF	100.00%	4.60 x 10^{4}
Escherichia coli 532			100.00%	1.10 x 10^{5}
Escherichia coli 502			99.82%	1.41 x 10^{5}
Escherichia coli 125			100.00%	5.70 x 10^{4}
Escherichia coli 636			100.00%	1.37 x 10^{5}
Escherichia coli 16			100.00%	8.45 x 10^{4}
Escherichia coli 110			100.00%	4.75 x 10^{4}
Escherichia coli 158			100.00%	6.30 x 10^{4}

Escherichia coli 452			100.00%	6.75 x 10^3
Escherichia coli 510			100.00%	6.55 x 10^4
Escherichia coli 663			100.00%	3.01 x 10^5
Escherichia coli 185			84.47%	1.31 x 10^4
Escherichia coli 515			100.00%	6.85 x 10^4
Escherichia coli 682			99.38%	2.40 x 10^4
Escherichia coli 108			100.00%	7.05 x 10^4
Escherichia coli 503			100.00%	1.10 x 10^5
Escherichia coli 632			100.00%	1.05 x 10^5
Escherichia coli 179			100.00%	1.72 x 10^4
Escherichia coli 201			100.00%	5.10 x 10^4
Escherichia coli 533			100.00%	3.85 x 10^4
Acinetobacter baumannii 145	Hospital strain, inpatient hospital	Cultural, morphological and biochemical properties, MALDI-TOF	98.72%	1.93 x 10^4
Acinetobacter baumannii 402			99.88%	4.20 x 10^4
Acinetobacter baumannii MA 65			99.81%	8.04 x 10^4
Acinetobacter baumannii 474			97.49%	1.17 x 10^4
Acinetobacter baumannii 869			98.73%	9.33 x 10^4
Acinetobacter baumannii Bor			98.54%	1.15 x 10^5
Acinetobacter baumannii PA			99.17%	8.33 x 10^4
Acinetobacter baumannii 67GKB			99.85%	3.35 x 10^4
Acinetobacter baumannii Gar	Hospital strain, intensive care unit	Cultural, morphological and biochemical properties	99.84%	9.14 x 10^4
Acinetobacter baumannii B-05	Hospital strain, inpatient hospital	Cultural, morphological and biochemical properties, MALDI-TOF	99.42%	2.55 x 10^4
Acinetobacter baumannii Ts 50-16	Hospital strain, intensive care unit	Cultural, morphological and biochemical properties	100.00%	1.53 x 10^5
Enterobacter sp. 1	Hospital strain, outpatient hospital	Cultural, morphological and biochemical properties, MALDI-TOF	99.77%	4.34 x 10^4
Enterobacter sp. 2			100.00%	1.41 x 10^4
Enterobacter sp. 3			100.00%	5.60 x 10^3
Enterobacter sp. 4			100.00%	1.59 x 10^4
Enterobacter sp. 5			100.00%	8.10 x 10^4
Enterobacter sp. 6			97.08%	4.32 x 10^4
Enterobacter sp. 7			98.97%	1.01 x 10^5
Enterobacter sp. 8			97.41%	1.70 x 10^4
Enterobacter sp. 9			99.71%	6.78 x 10^4
Enterobacter sp. 10			89.48%	2.60 x 10^4

*For all experiments, antimicrobial activity is shown as the mean value from three independent experiments. The antibacterial activity was expressed as follows: Antibacterial activity (%) = 100% - (CFU_{exp}/CFU_{cont})x100%, where CFU_{exp} is the number of bacterial colonies in the experimental culture plates, and CFU_{cont} is the number of bacterial colonies in the control culture plates. . "100%" Antibacterial activity means zero colonies on CFU_{exp} when plating 10 µl from initial experimental well. Antibacterial activity was arbitrarily regarded as meaningful when it was higher than 33%. CFU reduction value was obtained using the following equation: CFU reduction = CFU_{cont} - CFU_{exp}, where CFU_{exp} is the number of bacterial colonies in the experimental culture plates, and CFU_{cont} is the number of bacterial colonies in the control culture plates.

Table S3. Bacterial strains used to compare bactericidal activity of LysECD7-8his and LysECD7-SMAP. Antibacterial activity* of these proteins and CFU reduction* at concentration of protein upon treatment of 10^3 to 10^5 CFU/ml with 50 µg/ml.

Strain	Source	Method of Identification	Antibacterial Activity of LysECD7-8his	CFU Reduction	Antibacterial Activity of LysECD7-SMAP	CFU Reduction
Klebsiella pneumoniae Ts 104-14	Hospital strain, outpatient hospital	Cultural, morphological and biochemical properties, MALDI-TOF	46.23%	4.84×10^3	100%	1.05×10^4
Pseudomonas aeruginosa 12	Hospital strain, inpatient hospital		0%	0	100%	1.52×10^4
Pseudomonas aeruginosa 14			34.23%	4.45×10^3	100%	3.68×10^4
Staphylococcus aureus Z 73-14	Hospital strain, outpatient hospital		0%	0	99.91%	2.5×10^5
Staphylococcus haemolyticus G 58-0916			0%	0	92.33%	1.34×10^5

*For all experiments, antimicrobial activity is shown as the mean value from three independent experiments. The antibacterial activity was expressed as follows: Antibacterial activity (%) = 100% − $(CFU_{exp}/CFU_{cont}) \times 100\%$, where CFU_{exp} is the number of bacterial colonies in the experimental culture plates, and CFU_{cont} is the number of bacterial colonies in the control culture plates. "100%" Antibacterial activity means zero colonies on CFU_{exp} when plating 10 µL from initial experimental well. Antibacterial activity was arbitrarily regarded as meaningful when it was higher than 33%. CFU reduction value was obtained using the following equation: CFU reduction = $CFU_{cont} - CFU_{exp}$, where CFU_{exp} is the number of bacterial colonies in the experimental culture plates, and CFU_{cont} is the number of bacterial colonies in the control culture plates.

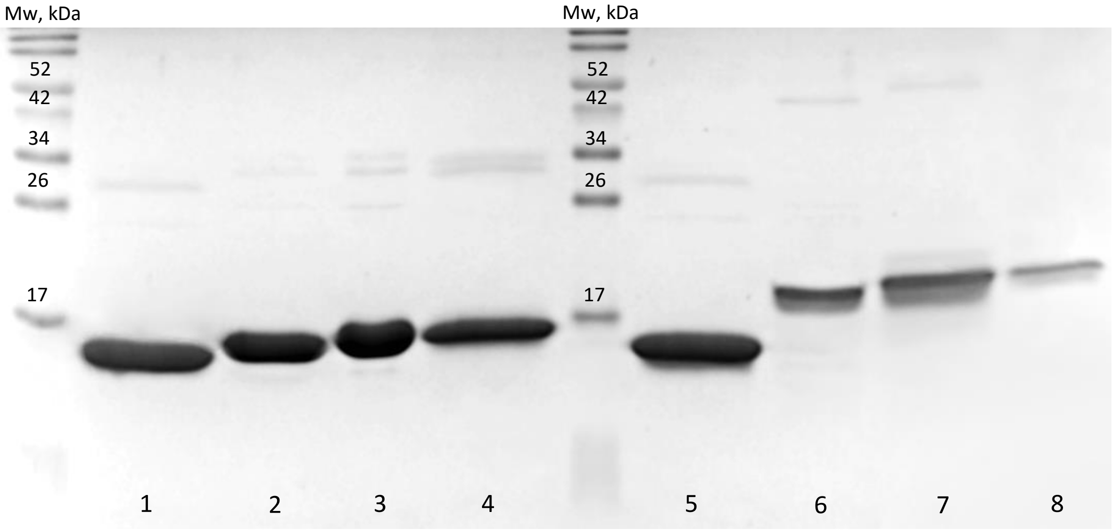

Figure S1. SDS-PAGE gel analysis of purified endolysins. 16% SDS-PAGE gel analysis of LysECD7 derivatives. Spectra™ Multicolor Broad Range Protein Ladder (Thermo Scientific, Vilnius, Lithuania) was used as a marker. 1 - LysECD7 (14.98 kDa), 2 - LysECD7-6his (15.7 kDa), 3 - LysECD7-8his (16.1 kDa), 4 - LysECD7-12his (16.5 kDa), 5 - LysECD7 (14.98 kDa), 6 - LysECD7-SMAP (17.18 kDa), 7 - LysECD7-flex-SMAP (18.16 kDa), 8 - LysECD7-rigid-SMAP (18.74 kDa).

A.

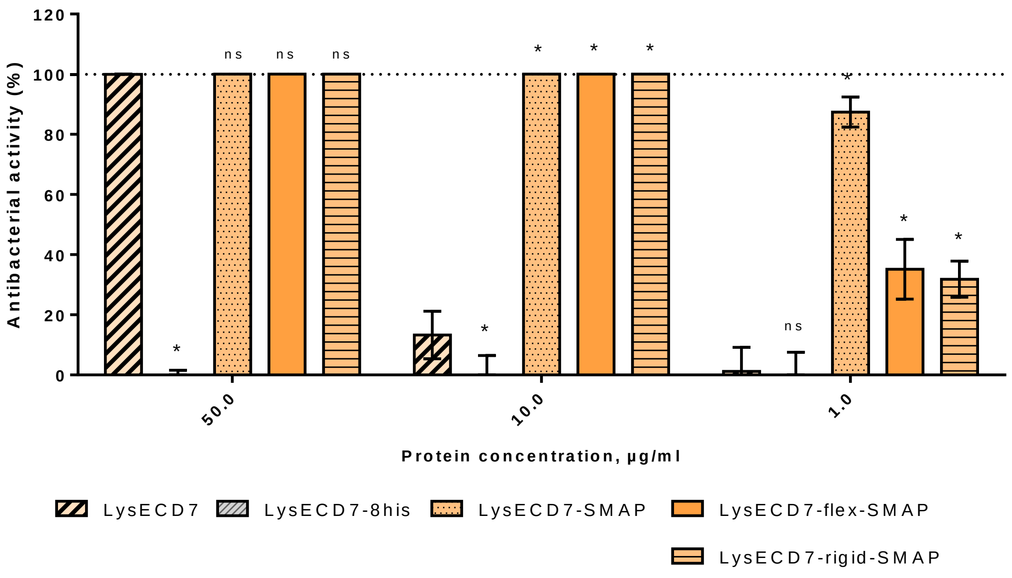

B.

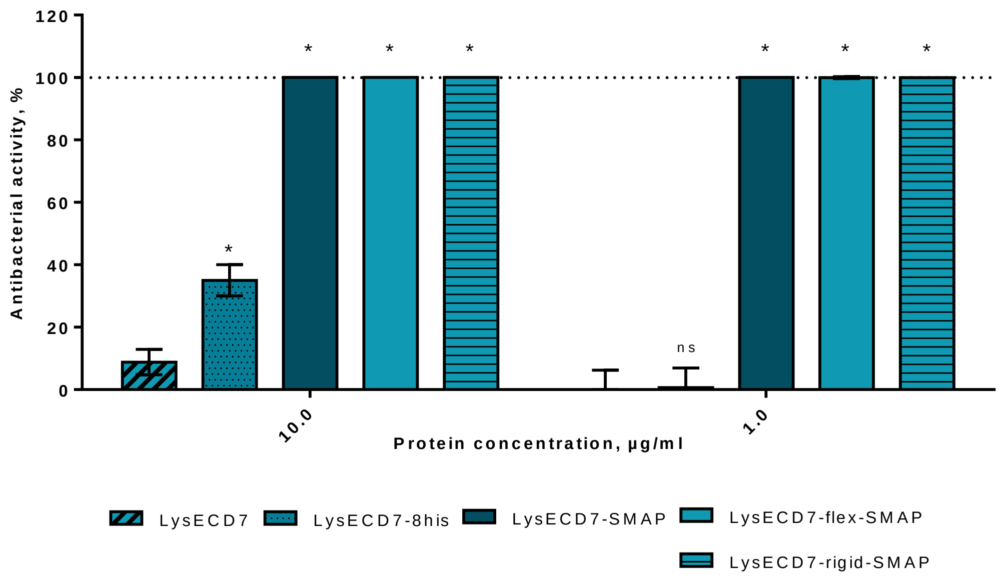

Figure S2. (**a**) Bactericidal activity of modified endolysins LysECD7 against *A. baumannii* Ts 50-16 in PBS buffer solution. For all experiments. the mean values are shown from three independent experiments (± standard deviation, SD). Asterisk (*) indicates significant effect on bactericidal activity comparing to LysECD7 enzyme ($p < 0.05$, one-way ANOVA, Dunnett's multiple comparisons test). (**b**) Efficiency of modified endolysins LysECD7 under different conditions. Bactericidal activity of modified endolysins LysECD7 against stationary-phase cells of *A. baumannii* Ts 50-16. For all experiments the mean values are shown from three independent experiments (± standard deviation, SD). Asterisk (*) indicates significant effect on bactericidal activity comparing to LysECD7 enzyme ($p < 0.05$, one-way ANOVA, Dunnett's multiple comparisons test).

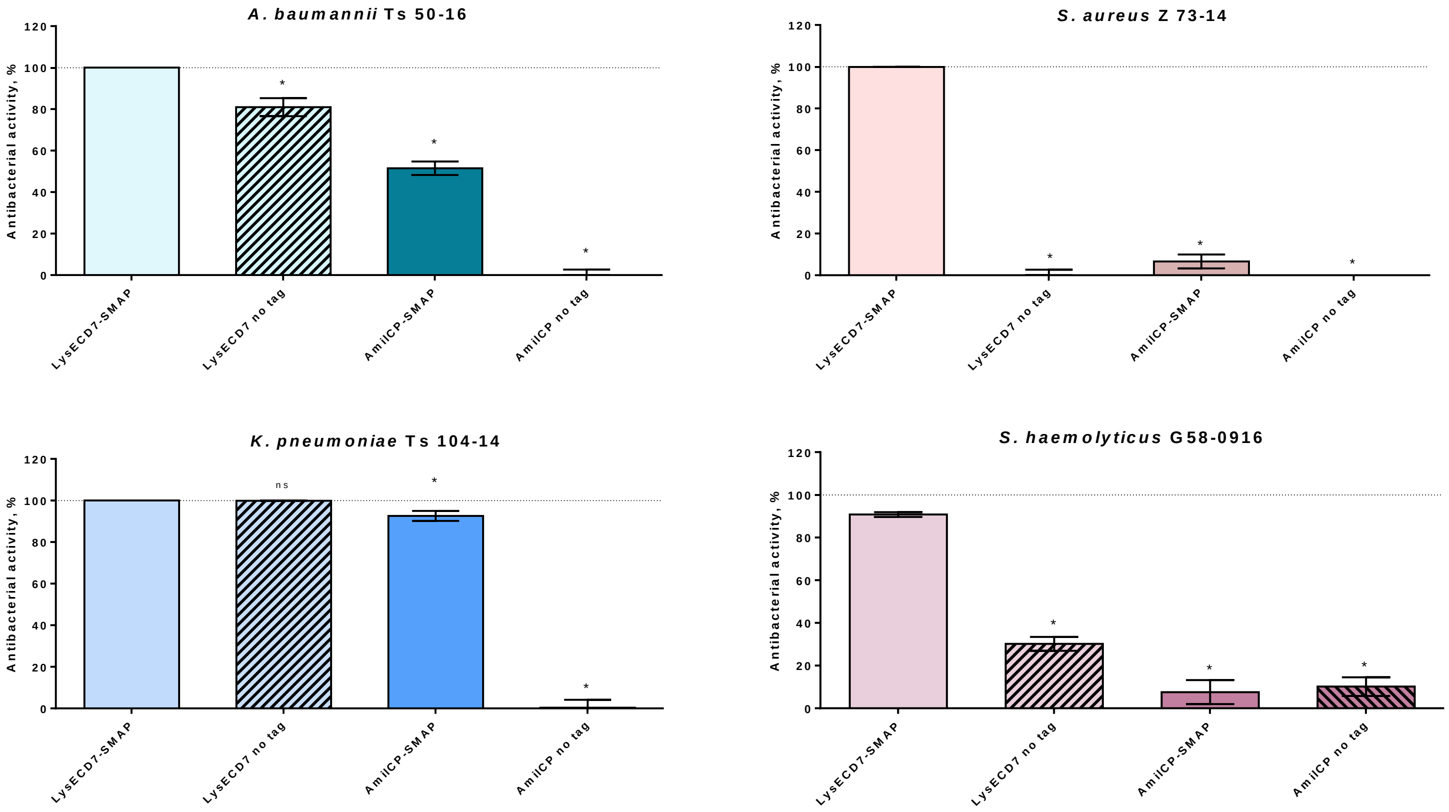

Figure S3. Influence of SMAP peptide introduction into the LysECD7 protein sequence on its antimicrobial activity. Bactericidal activity of LysECD7, its SMAP-modified version and SMAP peptide fused to a nonendolysin protein (AmilCP) against Gram-negative and Gram-positive isolates, diluted in 20 mM Tris HCl buffer pH 7.5. Proteins were assessed at following concentrations: 0.03 μM (corresponding to 0.5 μg/ml of LysECD7-SMAP protein) for *A. baumannii* Ts 50-16 and 2.9 μM (corresponding to 50.0 μg/ml of LysECD7-SMAP protein) for *Klebsiella* and *Staphylococcus* isolates. The culture dilution was 1 to 3 × 10^5 CFU/ml. For all experiments the mean values are shown from three independent experiments (± standard deviation, SD). Asterisk (*) indicates significant effect on bactericidal activity comparing to LysECD7-SMAP enzyme ($p < 0.05$, one-way ANOVA, Dunnett's multiple comparisons test).
